# Effect of Taste Recall Training in an Older Adult With Depression and Sarcopenia Presenting With Dysgeusia: A Case Report

**DOI:** 10.1111/ggi.70496

**Published:** 2026-04-17

**Authors:** Midori Miyagi, Uijin Park, Shunya Moue, Kan Goto, Motoyori Kanazawa, Shizuko Satoh, Toru Ogawa, Satoru Ebihara

**Affiliations:** ^1^ Department of Rehabilitation Medicine Tohoku University Graduate School of Medicine Sendai Japan; ^2^ Department of Psychosomatic Medicine Tohoku University Hospital Sendai Japan; ^3^ Division of Comprehensive Dentistry Tohoku University Graduate School of Dentistry Sendai Japan

Dear Editor,

1

Late‐life depression is closely associated with sarcopenia, with each condition exacerbating the risk of the other [1, 2]. Taste disorders (dysgeusia) have been reported to be associated with sarcopenia [3]. Patients with depression are susceptible to dysgeusia, possibly because of the depletion of serotonin and noradrenaline, which regulate taste recognition thresholds [4, 5]. Current treatments, primarily zinc supplementation, often fail. We previously developed taste recall training and demonstrated its efficacy in healthy adults [6]. Herein, we report the first clinical application of this method in an older patient with depression and sarcopenia, which resulted in improved taste and physical function.

A 76‐year‐old woman presented with a chief complaint of taste loss. The patient was initially referred to the Department of Comprehensive Dentistry at our hospital. Despite a healthy oral environment, she had low serum zinc levels (64.8 μg/dL), and zinc supplementation failed to improve her symptoms. As no clinical improvement was observed, psychological factors were suspected, and the patient was referred to the Department of Psychosomatic Medicine. She was diagnosed with mild depression, presenting mainly with dysgeusia, accompanied by anhedonia (loss of interest or pleasure) and depressed mood (self‐rating depression scale [SDS] score [7]: 46). She has been treated with pharmacotherapy (clonazepam 1.0 mg, escitalopram 10 mg) and psychotherapy; the dysgeusia persisted. Therefore, the patient was referred to our department for taste recall training. On referral to our department, history showed that the patient was widowed and lived alone. She maintained a regular exercise routine of 45 min of ergometer cycling daily and had no history of smoking. Physical findings revealed reduced grip strength (right: 15 kg, left: 14 kg) and the skeletal muscle index (SMI) measured by bioelectrical impedance analysis using an InBody S10 (InBody Co. Ltd., Seoul, Korea) was 5.2 kg/m^2^. Based on the AWGS 2025 diagnostic criteria [8], the patient was identified as having sarcopenia. Serum zinc levels were still low (62.6 μg/dL). Although re‐initiated supplementation normalized the zinc level (88 μg/dL), the subjective symptoms remained unchanged. Consequently, we initiated taste recall training alongside nutritional counseling and continued exercise. Nutritional counseling was performed once by a registered dietitian at the time of taste training initiation to assess her usual dietary habits. Although her overall dietary balance was adequate, considering her sarcopenic condition, we advised her to add one protein‐rich food to her lunch and to continue her regular exercise routine.

The training followed the protocol described by Otsubo et al. and used five basic tastes [6] (Figure 1a). The taste recognition threshold was defined as the concentration at which each taste quality could be recognized. The concentrations of taste solutions were prepared according to previous methods (Table S1). Sweet, salty, sour, and bitter solutions were prepared according to the taste disc method (Sanwa Kagaku Kenkyusho Co. Ltd., Nagoya, Japan), whereas the umami solution was prepared according to a previous study [9]. The program was conducted for three consecutive days. In Step 1, taste recognition thresholds were measured. In Step 2, the patient was exposed to a concentration one level above her threshold while being verbally informed of the taste and then matched to the threshold concentration. In Step 3, the patient memorized a concentration one level above the threshold and matched it with a concentration one level lower. In Step 4, the patient memorized the threshold concentration and matched it to a concentration that was one level lower. The assessment was conducted on the fourth day.

**FIGURE 1:**
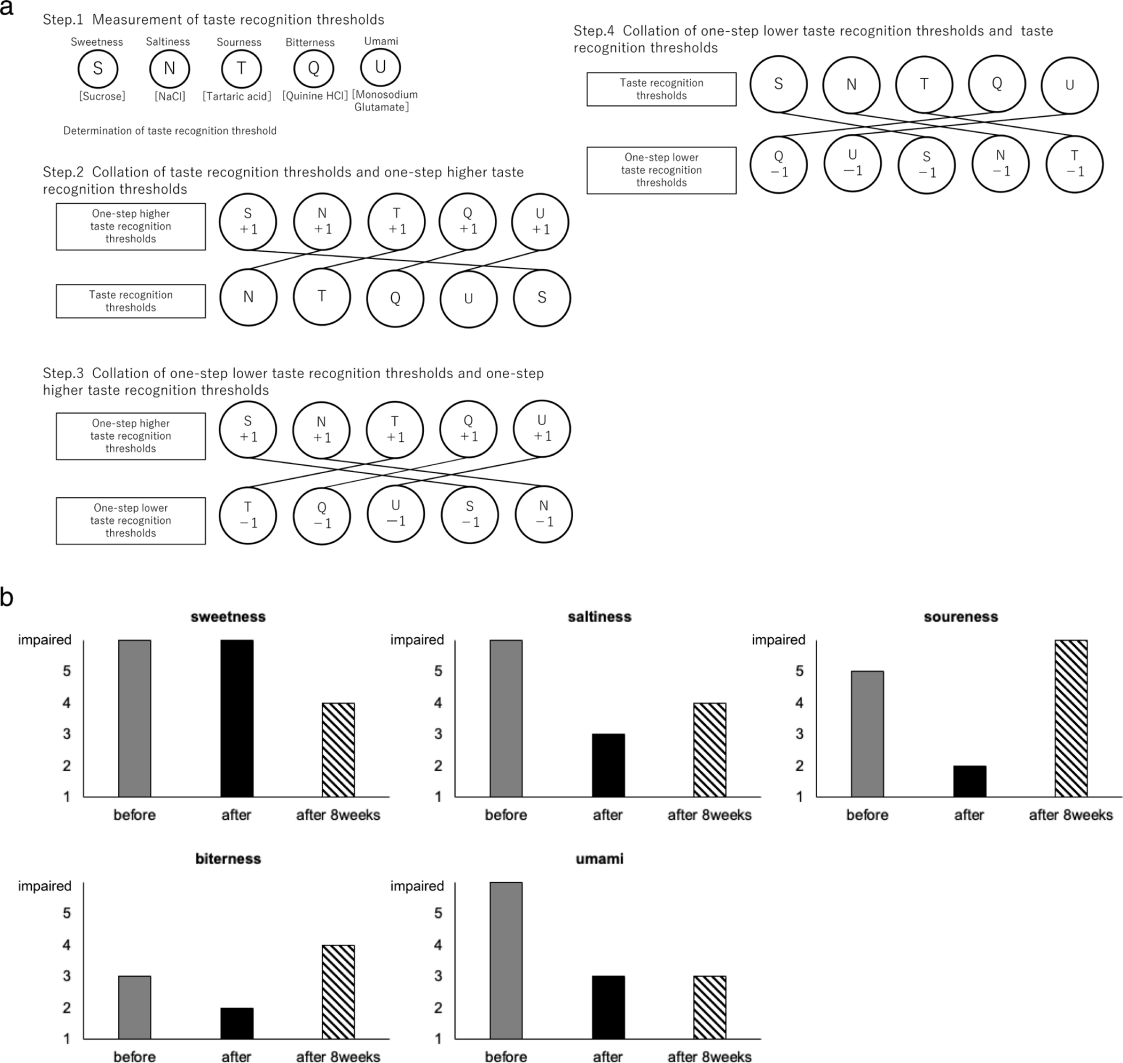
(a) Taste recall training method. Step 1: Measurement of taste thresholds for the five taste qualities. Step 2: Repeated exposure to the taste thresholds and concentrations one level above the threshold (+1), presented in random order. Step 3: Training with concentrations one level below (−1) and one level above (+1) the threshold, in random order. Step 4: Repeated exposure to the threshold and subthreshold (−1) concentrations, in random order. (b) Changes in the taste recognition thresholds of the patient.

Figure 1b shows changing of taste recognition threshold. At baseline, sweet, salty, and umami tastes were indistinguishable. By Day 4, the thresholds improved for all except sweetness. The patient subjectively reported regaining taste recognition. At 8 weeks, improvements were maintained, with the patient noting that the “Sweet foods taste delicious.” At the 6‐month follow‐up, the CNAQ‐J score [10] improved from 18 to 27 points. Her grip strength increased to 20 kg (right) and 19 kg (left), and she no longer met the criteria for sarcopenia, although the SMI remained stable (5.1 kg/m^2^). Despite the SDS score persisting at 50, the clonazepam dose was tapered to 0.5 mg as her mood had clinically stabilized.

In this case, zinc‐refractory dysgeusia improved following taste recall training. The improvement in taste recognition thresholds was predominantly attributed to the taste recall training, as nutritional guidance was provided only once, and no changes were made to her pre‐existing exercise regimen. Depression attenuates the response of the orbitofrontal cortex, a secondary taste cortex crucial for reward processing [11]. The initial generalized threshold elevation suggests such an impairment. We hypothesized that this training stimulates neural projections to the orbitofrontal cortex, reactivating taste and mood pathways. Although SMI showed no significant change, grip strength, a reflection of comprehensive physical function, improved substantially [12]. Since no resistance training specifically targeting grip strength was performed, this result seemed attributable to a comprehensive improvement in her psychosomatic state. The improvement in CNAQ‐J suggests that the recovered taste enhanced appetite. Additionally, medication reduction indicates a positive psychological impact. Limitations include the need for short‐term memory testing, potentially excluding cognitively impaired patients, and the burden of consecutive hospital visits.

Taste recall training improved taste thresholds and physical function and allowed medication reduction in an older patient with depression. This method may be a feasible approach for sensory rehabilitation.

## Author Contributions

Conceptualization: Satoru Ebihara and Midori Miyagi. Methodology: Satoru Ebihara and Midori Miyagi. Data acquisition: Midori Miyagi, Uijin Park, Shizuko Satoh, Shunya Moue, and Kan Goto. Writing – original draft preparation: Midori Miyagi. Writing – review: Motoyori Kanazawa, Toru Ogawa, and Satoru Ebihara. Drafting the work or revising it critically for important intellectual content. Final approval of the version to be published. Agreement to be accountable for all aspects of the work: All authors.

## Funding

This study was supported by the 30th Umami Research Grant from the Society for Research on Umami Taste, Japan to MM and the 3rd JGS Grant for Geriatric Nutrition Research supported to MM.

## Consent

Written informed consent was obtained from the patient for publication of this case report. The Medical Ethics Committee at Tohoku University approved the study protocol (No. 2023‐1‐868).

## Conflicts of Interest

The authors declare no conflicts of interest.

## Supporting information


**Table S1:** Concentrations of the five basic taste substance.

## Data Availability

Data supporting the findings of this study are available from the corresponding author upon reasonable request.
