# Supplementary material for: Effect of Taste Recall Training in an Older Adult With Depression and Sarcopenia Presenting With Dysgeusia: A Case Report
Source: Geriatr Gerontol Int. 2026 Apr 17;26:e70496. doi: 10.1111/ggi.70496 (PMC13090434; doi:10.1111/ggi.70496)
Supplement: Supplementary file 1 — Table S1: Concentrations of the five basic taste substance. [file GGI-26-0-s001.docx]

Table S1. Concentrations of the five basic taste substance

| Concentration level | Sweet  (Sucrose) | Saltiness  (Sodium chloride) | Sourness  (Tartaric acid) | Bitterness  (Quinine hydrochloride) | Umami  (Monosodium glutamate) |
| --- | --- | --- | --- | --- | --- |
| 1 | 8.8 | 51.4 | 1.3 | 0.025 | 1 |
| 2 | 74 | 214 | 13.3 | 0.5 | 5 |
| 3 | 292 | 856 | 133 | 2.5 | 10 |
| 4 | 584 | 1710 | 266 | 12.5 | 50 |
| 5 | 2336 | 3420 | 532 | 100 | 100 |

Values are expressed in millimolar (mM) concentration.
